# Immobility of the Lower Jaw

**Published:** 1846-10

**Authors:** R. L. Howard

**Affiliations:** Columbus, Ohio


*Immobility of the Lower Jaw.* By R. L. HOWARD, of Columbus, Ohio.—M. B. S., aged 9 years, on the 13th of May, 1846, was brought from Sunbury, Delaware co., O. for the purpose of consulting me respecting an immobility of his lower jaw. The history of the case is briefly as follows: Six years ago, when the boy was but three years old, he was attacked with some disease, for which the physician thought proper to administer calomel, which it is said he continued to exhibit for fourteen days in succession. The consequence was, a most violent ptyalism supervened, together with inflammation and sloughing of the inner portions of the cheeks, and oxfoliation of the alveolary processes of many of the molar teeth.—During the process of cicatrization, strong inelastic bands formed on both sides, but much the most extensive on the left. These bands were attached to the jaws above and below, and bound them firmly together. The integuments on neither side had ever been attacked by gangrene.

As the boy, though quite spare and delicate, seemed perfectly healthy, I resolved to overcome this apparent anchylosis, by an operation. Accordingly, with the assistance of Prof. Judkins of Cincinnati, and Prof. Butterfield of Willoughby, I proceeded at once to the undertaking. Being seated in a common chair, with his head thrown back and held by an assistant, Dr. Judkins insinuated a thin pine wedge between his incisor teeth, for the purpose of putting the parts upon the stretch and separating the jaws, whileI divided the connecting bands. I at first introduced my bistoury along the lower jaw, and with a few sawing motions separated the whole adventitious substance from it. Turning the instrument upwards, I divided the parts in the same manner from the upper jaw, both of which were now separated to the distance of half an inch. I now seized the apparently ligamentous cicatrix, which was attached alone to the inner surface of the cheek, and dissected it out entire. Turning my finger upon the right side, I found a band of less extent and firmness, which I freely divided by one or two incisions. I was not yet able to separate the jaws as widely as I desired. Passing my finger backward again on the left side, I found the anterior margin of the masseter muscle offered very firm resistance. I divided it from the jaw, as far as seemed to be necessary, when we were able to bring it down as far as we desired.

I now introduced a pine block something over an inch in thickness, between the edges of the incisor teeth; and placed the little fellow in bed.

*May 20th.* The case has done well. The block has been kept between the teeth three-fourths of the time, and I have no doubt, if the parts are kept upon the stretch most of the time until they are perfectly healed, the operation will be entirely successful.

If the case proves successful, I shall attribute the success to two things. 1st. The entire removal of the cicatrix ; and, 2d, to the permanent separation of the jaws until the parts were healed. Very many operations for the removal of this difficulty have, within my observations, proved unsuccessful, and I apprehend that they terminated thus unfortunately, from a neglect of the very procedures which I consider of so much importance.

